# Targeted Gene Editing in Porcine Spermatogonia

**DOI:** 10.3389/fgene.2020.627673

**Published:** 2021-01-28

**Authors:** Dennis Webster, Alla Bondareva, Staci Solin, Taylor Goldsmith, Lin Su, Nathalia de Lima e Martins Lara, Daniel F. Carlson, Ina Dobrinski

**Affiliations:** ^1^Recombinetics, Inc., St. Paul, MN, United States; ^2^Department of Comparative Biology and Experimental Medicine, University of Calgary, Calgary, AB, Canada

**Keywords:** pig, spermatogonia, gene targeting, CRISPR/Cas9, homology directed repair, homology-mediated end joining

## Abstract

To study the pathophysiology of human diseases, develop innovative treatments, and refine approaches for regenerative medicine require appropriate preclinical models. Pigs share physiologic and anatomic characteristics with humans and are genetically more similar to humans than are mice. Genetically modified pigs are essential where rodent models do not mimic the human disease phenotype. The male germline stem cell or spermatogonial stem cell (SSC) is unique; it is the only cell type in an adult male that divides and contributes genes to future generations, making it an ideal target for genetic modification. Here we report that CRISPR/Cas9 ribonucleoprotein (RNP)-mediated gene editing in porcine spermatogonia that include SSCs is significantly more efficient than previously reported editing with TALENs and allows precise gene editing by homology directed repair (HDR). We also established homology-mediated end joining (HMEJ) as a second approach to targeted gene editing to enable introduction of larger transgenes and/or humanizing parts of the pig genome for disease modeling or regenerative medicine. In summary, the approaches established in the current study result in efficient targeted genome editing in porcine germ cells for precise replication of human disease alleles.

## Introduction

Applicable preclinical models are needed to investigate the pathophysiology of human diseases, develop novel treatments and medical devices, and improve approaches for regenerative medicine. While rodent models are currently the standard for early preclinical studies, pigs are physiologically, anatomically, and genetically more similar to humans than are mice and are delivering increasing value to biomedical research. Genetically modified pigs, such as pig models of cystic fibrosis (Rogers et al., 2009), neurofibromatosis type I (Isakson et al., 2018), and diabetes (Kleinwort et al., 2017), are essential where rodent models fail to recapitulate the full pathophysiological spectrum of a disease. The generation of biomedical pig models primarily relies on somatic cell nuclear transfer (SCNT) using cells genetically modified with engineered nucleases such as Zinc Finger nucleases (ZFNs), Transcription activator-like Effector Nucleases (TALENs), and Clustered Regularly Interspaced Short Palindromic Repeats/CRISPR-associated-9 (CRISPR/Cas9) (Tan et al., 2012).

Even though SCNT is a well-established process, it is inefficient and associated with abnormal fetal and placental development and neonatal mortality due to incomplete reprogramming of the somatic cell nuclei (De Sousa et al., 2001; Hill et al., 2002). Microinjection and electroporation of TALENs, ZFNs, and CRISPR/Cas9 into *in vitro* fertilized pig zygotes have been used to more efficiently produce gene-edited piglets that are free of SCNT (reprogramming)-associated defects (Armstrong et al., 2006; Bonk et al., 2008; Tian et al., 2009). However, microinjection and electroporation of engineered nucleases often result in genetic mosaicism that requires the time-consuming process of outcrossing of mutants to generate isogenic animals to investigate alleles of interest. Moreover, all of the current approaches for generating pig models require expensive specialized equipment and considerable expertise and time. An alternative approach for generating genome-edited animals is through the use of spermatogonial stem cells (SSCs) (Hamra et al., 2002; Orwig et al., 2002). SSCs, a subpopulation of undifferentiated type A spermatogonia, are unipotent stem cells that reside in the stem cell niche at the basement membrane of seminiferous tubules where they undergo a highly coordinated process of self-renewal and differentiation to form sperm (De Rooij, 2001). Hence, SSCs are the genetic basis of future generations. When cell populations containing SSCs are transplanted to a recipient testis, SSCs establish donor-derived spermatogenesis, making them an ideal target for genetic modification (Brinster and Avarbock, 1994; Brinster and Zimmermann, 1994). Currently, there are no molecular markers that allow prospective identification of SSCs within the population of undifferentiated type A spermatogonia.

Engineered nucleases have been utilized in cultured mouse and rat spermatogonia to produce progeny with targeted gene knockout or gene-corrected alleles after transplantation of gene-edited SSCs and *in vitro* fertilization or natural breeding (Chapman et al., 2015; Sato et al., 2015; Wu et al., 2015). Although viral-mediated transgenesis and transplantation of pig spermatogonia containing SSCs resulted in transgenic embryos after *in vitro* fertilization (Zeng et al., 2013), the lack of site-specific targeting due to random integration and use of viral vectors limits the application of this approach for production of biomedical pig models. Introduction of site-specific TALENs in pig spermatogonia using nucleofection resulted in non-homologous end joining (NHEJ) with indel efficiencies of up to 18% but at the expense of low cell viability (Tang et al., 2018). Moreover, optimization of gene-editing efficiency with cell viability was insufficient to facilitate homologous recombination when a single-strand oligo donor (ssODN) repair template was introduced with the TALENs (unpublished). Here we report the site-specific genetic engineering of porcine spermatogonia using the CRISPR/Cas9 ribonucleoprotein (RNP) system resulting in efficient generation of custom indel and single nucleotide polymorphism (SNP) alleles through homologous recombination of an ssODN repair template. In addition, we demonstrate integration of a ubiquitin-driven EGFP cassette/transgene into the safe harbor *ROSA26* locus of spermatogonia using CRISPR/Cas9 RNP and plasmid donors linearized within the cell to provide a template for homology-mediated end joining (HMEJ) repair.

## Materials and Methods

### Isolation and Enrichment of Germ Cells

Testes were obtained from 8-week-old pigs by surgical castration. Single-cell suspensions were prepared by a sequential enzymatic digestion protocol (Sakib et al., 2019). Briefly, the tunica albuginea and visible connective tissue were removed, and the exposed seminiferous tubules were dissociated with Type IV collagenase (2 mg/ml; Sigma–Aldrich Cat# C5138, RRID:AB_008988) in Dulbecco modified Eagle medium (DMEM, Sigma–Aldrich Cat# D6429, RRID:AB_008988) at 37°C for 20–40 min with occasional agitation, followed by incubation at 37°C for 30 min in DMEM with Type IV collagenase (2 mg/ml; CEDARLANE Laboratories Limited LS004189, RRID:AB_004462) and hyaluronidase (1 mg/ml; Sigma–Aldrich Cat# H3506, RRID:AB_008988). The digested tubules were rinsed three times in Dulbecco phosphate-buffered saline (DPBS, Ca^2+^ and Mg^2+^ free; Sigma–Aldrich Cat# D8537, RRID:AB_008988) and further digested with 0.125% (w/v) trypsin and 0.5 mM ethylenediaminetetra-acetic acid (EDTA) (Sigma–Aldrich Cat# T4049, RRID:AB_008988) at 37°C for 15–20 min. DNase I (7 mg/ml in DMEM; Sigma–Aldrich Cat# DN25, RRID:AB_008988) was added during the digestion process as needed. After trypsin digestion, the cell suspension was filtered through 70 μm and 40 μm cell strainers sequentially (BD Biosciences). The single cells were then collected by centrifugation at 500 *g* for 5 min at room temperature (RT) and the cell pellet was resuspended in DMEM/F-12 (Life Technologies Cat# 11330032, RRID:AB_008817) with 5% fetal bovine serum (FBS, Life Technologies Cat # 12483020, RRID:AB_008817) for differential plating.

### Differential Plating

Immediately after tissue digestion, 2.5 × 10^7^ cells in 8 ml DMEM/F-12 with 5% FBS were plated onto 100 mm tissue culture plates and incubated at 37°C in 5% CO_2_. Three sequential rounds of differential plating were performed (1.5 h, 1 h, and overnight). At the second and third round of plating, cell suspensions from two plates were combined and plated onto a new 100 mm culture plate. Attached cells were discarded. After overnight incubation, supernatant from all the plates was pooled. To collect loosely adhered germ cells, 2–3 ml of diluted Trypsin/EDTA (1:5 or 1:20 dilution with PBS) was added to each plate. Plates were incubated at 37°C for 2 min and then at RT for 3 min with constant agitation to release attached germ cells without disturbing somatic cells. The reaction was stopped by adding an equal volume of DMEM/F12 with 10% FBS. Cell suspensions were pooled from all plates, combined with cells collected from the supernatants, pelleted by centrifugation at 500 *g* for 5 min, and washed twice with PBS. After washing, cells were plated again onto 100 mm plates in DMEM/F12 with 5% FBS for 8 min at RT, and cell debris and contaminating red blood cells were gently and slowly collected from the top and discarded, and GSCs were collected from the bottom of the plates.

### Fluorescence-Activated Cell Sorting (FACS)

**FIGURE 1:**
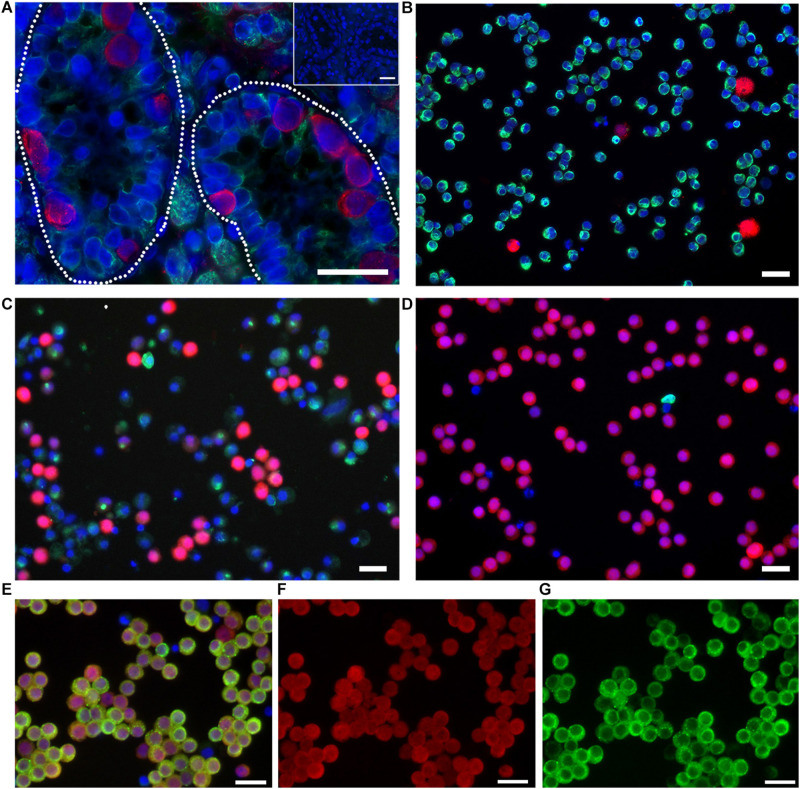
Undifferentiated spermatogonia express UCH-L1. **(A)** Testis tissue from 8-week-old pig. Broken line outlines seminiferous tubules. Inset: negative control. **(B)** Testicular cells after enzymatic digestion of testis tissue. **(C)** Enriched spermatogonia after differential plating. **(D–F)** Highly enriched spermatogonia after FACS for light scatter properties. UCH-L1 red, vimentin green** (A–D)**, DDX4 green **(E,G)**, DAPI blue, bars = 25 μm.

Germ cells were further enriched by sorting for light scatter properties as described (Tang et al., 2018). Briefly, enriched cell fractions collected after differential plating containing 58.6 ± 0.61% UCH-L1 + spermatogonia (mean ± SEM, *n* = 3; Figure 1C) were resuspended in PBS with 1% BSA (Sigma–Aldrich Cat# A7906, RRID:AB_008988) and subjected to sorting on a FACSAria III (Becton Dickinson, BD FACSARIA III cell sorter, RRID:AB_016695). A gate was drawn around the distinctive germ cell population on the forward and side light scatter dot plot, and cells within this gate were sorted. Sorted cells were pelleted and washed once with PBS. The viability of sorted cells was assessed by Trypan Blue staining. A sample was fixed with 2% paraformaldehyde (PFA; Thermo Fisher Scientific Cat# 41678-5000, RRID:AB_008452) and assessed for enrichment by immunocytochemistry with antibodies against UCH-L1 and Vimentin. UCH-L1 is a spermatogonia-specific marker that was used to assess the enrichment efficiency and to determine the percentage of germ cells present in a given cell population (Luo et al., 2009; Figure 1). Vimentin was used to label somatic cells. For each sorting experiment, 1000 cells were evaluated. As reported previously (Tang et al., 2018), cells enriched by fluorescence-activated cell sorting (FACS) contained 88.7 ± 4.36% UCH-L1 + spermatogonia (mean ± SEM, *n* = 3; Figures 1D–F).

### Immunocytochemistry

Cells were fixed in 2% PFA for 30 min at RT and washed twice with PBS. Cells were then transferred onto slides for immunostaining by cytospin centrifugation (800 *g* for 5 min at RT) (Thermo Fisher Scientific Cat# A78300002, RRID:AB_008452), permeabilized in PBS with 0.1% Triton-X (EMD4Biosciences Cat# 9410, RRID:AB_008441), and washed three times in PBS prior to 1 h blocking with 3% BSA. Cells were incubated with the following primary antibodies overnight at 4°C: rabbit-anti-human UCH-L1 (Abcam Cat# ab108986, RRID:AB_10891773) at 1:500, mouse anti human DDX4 (Abcam Cat# ab27591, RRID:AB_11139638) at 1:100, and mouse-anti-pig vimentin-Cy3 at 1:400 (Sigma–Aldrich Cat# C9080, RRID:AB_259142). Three washes were performed after overnight primary antibody incubation and secondary antibodies donkey-anti-mouse IgG Alexa Fluor 488 (1:1000) or donkey-anti-rabbit IgG Alexa Fluor 594 (1:1000) were added onto samples. After 1 h RT incubation, cells were washed three times and mounted in VECTASHIELD Mounting Medium with DAPI (Vector Laboratories Cat# H1200, RRID:AB_000821) for imaging. For each cell prep experiment, images from five to six randomly chosen fields were collected and >1000 cells were evaluated.

### CRISPR Design and RNP Complexing

CRISPR gRNAs were designed using Cas-Designer (CRISPR RGEN Tools; Bae et al., 2014; Park et al., 2015), selected for minimal predicted off-target sites, and purchased as Alt-R^®^ CRISPR-Cas9 crRNAs with Alt-R^®^ CRISPR-Cas9 tracrRNA from Integrated DNA Technologies (IDT) (Coralville, IA, United States) or as sgRNAs from Synthego (Redwood City, CA, United States). Guide RNAs for each locus are listed in Table 1. Cas9 protein, Alt-R^®^ S.p. HiFi Cas9 Nuclease V3, or sNLS-SpCas9-sNLS Nuclease were purchased from IDT (Coralville, IA, United States) or Aldevron (Madison, WI, United States), respectively. To anneal the crRNA and tracrRNA, equimolar concentrations of each were combined and heated to 95°C for 5 min and then cooled to 22°C at −0.1°C/s. To form RNP complexes, crRNA:tracrRNA duplex was incubated at a ratio of 1.14:1 with Cas9 protein and incubated at RT for 10–15 min.

**TABLE 1:** Guide RNAs, ssOligos, and primers for HDR at three loci.

Locus	Guide RNA (5′–3′)	HDR oligo (5′–3′)	Primers (5′-3′)	Amplicon size (bp)	Cut band sizes (bp)
*HNF1a*	ssHNF1a g4.3 GGCGCAAGGA AGAAGCAUUU	ssHNF1A g4.3 HD3-KO GTCTACAACTGGTTTGCCAATC GGCGCAAGGAAGAAGCATAAA GCTTTTTCGGCACAAGTTGGC CATGGACACGTACAGTGGGCC ACC	ssHFN1A E4 NJ F3: GAGGGTTCTTCTGTGCCTGG ssHFN1A E4 NJ R3: GAGTGGAGAAAGCCAGGAGG	NHEJ: 415 HDR: 423	166 + 249 171 + 252
*ROSA26*	ssROSA g2 GGAUUUUUCU AGGCCCAGGG	ssROSA g2 HD3 ATGACGAGATCGCGGGGGAG GGAGGGATTTTTCTAGGCCAT AAAGCTTGGGCGGTCCTTAGG AAAAGGAGGCAGCAGAGAAC TCCCATA	ssROSA g2 F2: GCCTGAAGGACGAGACTAGC ssROSA g2 R2: AACACGCAGTCTCAATGCAT	NHEJ: 530 HDR: 538	254 + 276 257 + 281
*INS*	ssINS g2: CUGGUAGAGG GAACAGAUGC	ssINS g2 C94Y *Spe*I CCTAGTSDTGCAGTAGTTCTCC AGCTGGTAGAGGGAACAGATA CTAGTGTAGCACTGCTCCACG ATGCCACGCTTCTGCGGGGGC CCCTCC	ssINS E3 NJ F2: GTGGCTGTCTCTGTGTGACC ssINS E3 NJ R2: GGAAGCTTAGAGCAGCCGAT	NHEJ: 361 HDR: 361	136 + 225 132 + 229

### Germ Cell Nucleofection

Nucleofection was performed with the Amaxa Nucleofector II device (Lonza Cat# AAD-1001S RRID:AB_000377) essentially as described (Tang et al., 2018). Enriched cells were resuspended in solution V and transfected with the program X-005. Each transfection included RNP complexes formed by incubation of 200 pmol guide RNA with 175 pmol Cas9 protein for 15 min at RT. RNP complexes were introduced to 1 × 10^6^ cells and transferred onto six-well plates in αMEM Advanced culture medium (Life Technologies Cat# 12492013, RRID:AB_008817) supplemented with 1% FBS, 0.1% BSA, 1X non-essential amino acids (Life Technologies Cat# 11140-050, RRID:AB_008817), 1 mM sodium pyruvate (Life Technologies Cat# 11360-070, RRID:AB_008817), 15 mM HEPES (Life Technologies Cat# 15630-080, RRID:AB_008817), 2 mM L-glutamine (Life Technologies Cat# 25030-081, RRID:AB_008817), 10 μM beta-mercaptoethanol (Sigma–Aldrich Cat# M7522, RRID:AB_008988), 100 U/ml Penicillin-100 μg/ml Streptomycin (Sigma–Aldrich Cat# P4333, RRID:AB_008988), and 10 ng/ml glial cell line-derived neurotrophic factor (GDNF; R&D systems Cat# 212-GD-010, RRID:AB_006140) for cell recovery and short-term cell culture. After overnight recovery, the medium was replaced with fresh culture medium, and cells were incubated at 30 or 37°C for 3–5 days depending on the experimental design. At the end of incubation, cells were harvested by gentle trypsinization (1:5 dilution of 0.25% Trypsin/EDTA). The number of cells collected was counted by hemocytometer and the viability was assessed by Trypan Blue (Thermo Fisher Scientific Cat#15250061, RRID:AB_008452) staining. Collected cells were used for further analysis.

### Transfections With ssODNs

Single-stranded DNA templates for homology directed repair (HDR) were manufactured by IDT, Coralville, IA, United States, selecting the 100 nmol synthesis and standard desalting options. Transfections with ssODNs were performed as above including 168 pmol of ssODN template specific for each gene. Single-stranded DNA templates for each locus are listed in Table 1.

### HMEJ Transfections

For HMEJ insertion, enriched germ cells were transfected with Universal and *ROSA26* RNP (Table 2 and Supplementary Figure S1) complexes in the quantities indicated above, along with 1.7 μg of the eGFP plasmid cassette and electroporation enhancer (IDT, Coralville, IA, United States, Cat# 1075916).

**TABLE 2:** Guide RNAs and primers for HMEJ.

Name	Sequence (5′–3′)	Purpose
Universal gRNA	GGGAGGCGUUCGGGCCA CAG	Liberate repair cassette from plasmid
ss*ROSA26* g2 F2	GCCTGAAGGACGAGACT AGC	5′ Junction screening (585 bp amplicon)
ss*ROSA26* HDR Test R3	GAGATCCCTCCGCAGAA TCG	
bt*ROSA26* Ins F1	CACATGGTCCTGCTGGA GTT	3′ Junction screening (589 bp amplicon)
ss*ROSA26* g2 R2	AACACGCAGTCTCAATG CAT	

### Assessment of Targeted Mutagenesis and Homology Directed Repair

Genomic DNA was extracted from spermatogonia using PCR-safe lysis buffer [10 mM Tris-Cl, pH 8.0; 2 mM EDTA; 2.5% (vol/vol) Tween20; 2.5% (vol/vol) Triton-X 100; 100 mg/ml Proteinase K (Sigma–Aldrich Cat# P2308, RRID:AB_008988)] followed by incubation at 50°C for 60 min and 95°C for 15 min. The genomic region flanking the gRNA target site was PCR amplified with gene-specific primers (Table 1) and AccuStart^TM^ Taq DNA Polymerase HiFi (QuantaBio, Cat# 95085, Beverly, MA, United States) according to the manufacturer’s recommendations. To analyze the frequency of NHEJ mutation in a population, the Surveyor mutation detection kit (Cat# 706020; IDT) was used according to the manufacturer’s recommendations using 10 μl of the PCR product as described above. To analyze the frequency of HDR mutations in a population, restriction endonuclease digest was performed. Briefly, 6 μl of PCR product was digested with 6–10 units of enzyme, *ROSA26* and *HNF1a-Hin*dIII, *INS-Spe*I, in recommended buffer (Cat # R3144 and R0133, New England BioLabs, Ipswich, MA, United States; RRID:AB_013517). Surveyor and restriction digest reactions were resolved on a 10% TBE polyacrylamide gels and visualized by ethidium bromide (Fisher Scientific Cat# BP102-5) staining. Densitometry measurements of the bands were performed using ImageJ (ImageJ, RRID:AB_003070). The mutation rate of Surveyor reactions was calculated as described previously (Guschin et al., 2010) and the HDR rate of the restriction digest reactions was calculated as [(sum of RFLP bands/sum of wildtype and RFLP bands)^∗^100].

### Analysis of HMEJ Insertions

After genomic DNA extraction from GFP positive and negative GSC-enriched populations, the 5′ and 3′ junctions from the endogenous gene to the exogenous cassette were PCR amplified with 2X AccuStart^TM^ II PCR Supermix (Cat# 95136, QuantaBio) according to the manufacturer’s recommendations. Universal gRNA and primer sequences for the 5′ and 3′ junctions are shown in Table 2. PCR products were resolved on an agarose gel and products in the region of the precise integration expected product sizes (5′ junction 585 bp, 3′ junction 589 bp) were excised and gel purified with the Qiagen Gel Extraction Kit (Qiagen, Cat# 28706, RRID:AB_008539) per the manufacturer’s instructions. Purified PCR products were TOPO cloned into the pCR4-TOPO sequencing vector (ThermoFisher Scientific Cat#K457502, RRID:AB_008452); 5–20 clones per junction were sequenced via Sanger sequencing.

### Statistical Analysis

Data were analyzed using GraphPad Prizm 4.0 (GraphPad Software Inc., La Jolla, CA, United States; RRID:AB_000306). A Student’s *t*-test and ANOVA were performed to compare groups. Data were expressed as means ± SEM, and *P* < 0.05 was considered significant.

### Bioethics

All animal experimentation was conducted with approval and oversight of the Animal Care and Use Committee at the University of Calgary.

## Results

### Germ Cell Gene Editing by HDR

**FIGURE 2:**
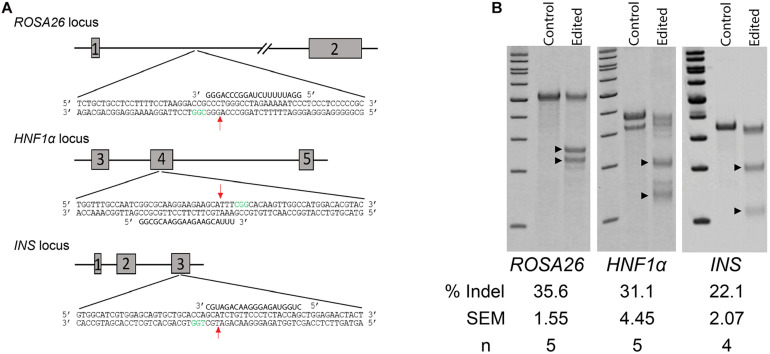
Non-homology mediated end joining (NHEJ) in germ cells edited with CRISPR/Cas9 RNPs. **(A)** Schematic of targeted loci and guide RNAs. Red arrows indicate location of double-strand breaks (DSBs). Green nucleotides represent the Protospacer Adjacent Motive (PAM). **(B)** Efficiency of indel formation (NHEJ) at three targeted loci. Arrow heads indicate digested fragments (see Table 1).

As the initial live offspring produced by GST would be heterozygote due to modification of the male germline only, we chose to evaluate gene editing at three biomedically relevant loci associated with either dominant forms of diabetes mellitus, *Hepatocyte nuclear factor-1 alpha* (*HNF1a*) and *insulin* (*INS*), or a common safe-harbor locus for transgene insertion, *ROSA26* (Figure 2A). Initially, we compared gene-editing efficiency of two gRNA structures, duplexed and single guides, delivered as RNP complexes followed by culture at either 30 or 37°C. We found that neither guide structure nor temperature had an effect on indel formation by Surveyor assay at two loci, *HNF1a* (not shown) or *ROSA26* (Supplementary Figure S1). Next, we evaluated the efficiency of indel formation at all three loci and found editing ranging from 20 to 35%, depending on locus (Figure 2B). Of note, cell recovery at 37 versus 30°C was slightly higher (82.8 ± 2.73 versus 76.9 ± 0.61%, *n* = 6, *p* < 0.5), and since temperature did not influence editing efficiency by CRISPR/Cas9 RNPs, further studies were performed using recovery at 37°C.

**FIGURE 3:**
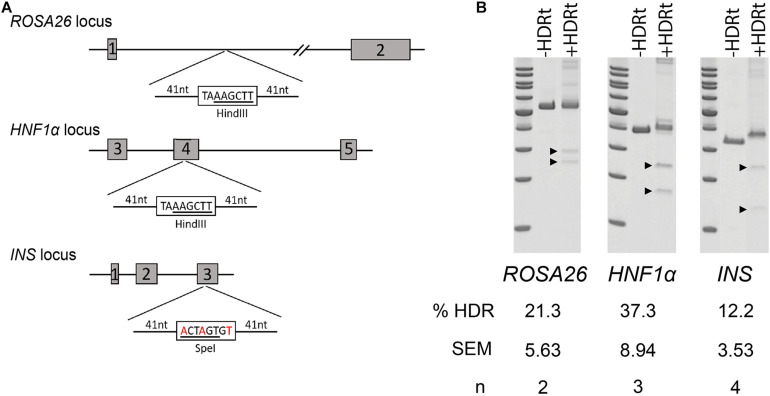
Homology directed repair (HDR) in germ cells edited with CRISPR/Cas9 RNPs. **(A)** Schematic of targeted loci with HDR templates and restriction sites. Boxed nucleotide sequences represent insertions in the *ROSA26* and *HNF1a* loci, and red nucleotides represent SNPs in the *INS* locus. **(B)** Efficiency of HDR measured by restriction fragment length polymorphism (RFLP) analysis at three targeted loci. ±HDRt indicates transfection with or without the HDR template. Arrow heads indicate cut fragments (see Table 1).

The high recovery and efficiency of CRISPR/Cas9 RNP mediated editing in spermatogonia provided the basis for more complex modifications. To test this, we designed a series of 90-mer ssODN templates to stimulate homology-directed repair (HDR) (Figure 3A). The templates for *ROSA26* and *HNF1a* were designed to insert a novel 8-base-pair sequence containing a *Hin*dIII restriction endonuclease site, intended to replicate a premature termination codon as observed in common maturity onset diabetes of the young (MODY) alleles. For the *INS* gene, we designed the template to replicate the C96Y mutation (C94Y in pigs; Renner et al., 2013) known to cause permanent neonatal diabetes mellitus (PNDM) in humans, a type 1 diabetes like disease. In contrast to the insertion designs of *HNF1a* and *ROSA26*, the *INS* repair template introduces three SNPs to alter a codon, mutate the protospacer motif to prevent re-cleavage of homology repaired sequences, and introduce a silent *Spe*I restriction endonuclease site (Figure 3A). Each of the templates was delivered into spermatogonia by CRISPR/Cas9 RNPs using the conditions used for NHEJ. The efficiency of HDR was measured by restriction fragment length polymorphism (RFLP) analysis revealing robust HDR at each locus (Figure 3B). As observed previously (Tan et al., 2013), the insertion HDR alleles, *HNF1a* and *ROSA26*, was more efficient than the SNP allele, *INS*.

### Germ Cell Gene Editing by HMEJ

**FIGURE 4:**
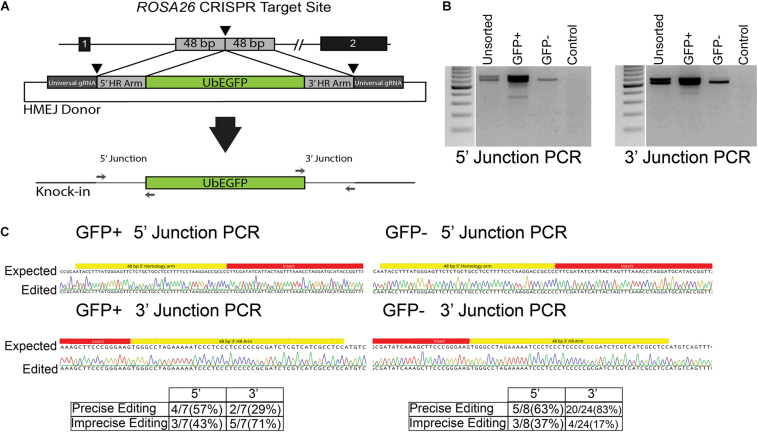
Homology mediated end joining (HMEJ) in germ cells edited with CRISPR/Cas9 RNPs. **(A)** Schematic of HMEJ at the *ROSA26* locus with the UbEGFP donor. The genome is cut with *ROSA26* gRNA while the donor template is liberated from a plasmid with the universal gRNA. **(B)** HMEJ-mediated knock-in of the UbEGFP reporter into the *ROSA26* locus with CRISPR/Cas9 RNPs induced genomic double-strand breaks (DSBs). PCR analysis at 11 days revealed 5′ and 3′ integration junctions in the unsorted, GFP+ and GFP- germ cell populations but no non-DSB stimulated integration of the ubiquitin-EGFP donor (Control). **(C)** Sequencing of junction amplicons at 11 days indicated precise and imprecise HMEJ in GFP+ and GFP- sorted germ cells.

While efficient, HDR with ssODN templates is limited to creation of small (<50 bp) changes to the genome. To expand the utility of GSC editing, we evaluated whether HMEJ could be used to integrate cargo into the *ROSA26* safe harbor locus. The plasmid template was designed to integrate an eGFP expression cassette under control of the ubiquitin C (UbC) promoter (Figure 4A and Supplementary Material). The cassette was designed based on the pGTag vector series with universal gRNA target sites with no predicted off targets in the swine genome and short, 48-base-pair homology arms flanking the insertion cassette (Figure 4A) (Wierson et al., 2020). When the cassette is introduced into cells with CRISPR/Cas9 RNPs targeted to the universal gRNA site, the insertion cassette is liberated from the plasmid and integrated into the cut target site via a HMEJ mechanism. After transfection of enriched spermatogonia with each component, the cells were cultured at 37°C and sampled at 4 and 11 days post transfection, the latter time point to reduce non-integrated transient eGFP expression prior to FACS. FACS analysis revealed that about 8% of spermatogonia stably expressed eGFP after 11 days in culture (13.9 ± 3.77% eGFP+ cells after 4 days in culture, and 7.9 ± 1.07% eGFP+ cells after 11 days in culture; mean ± SEM, *n* = 3). Molecular analysis was performed on populations of cells 4 and 11 days post transfection. PCR junction products were observed for the 5′ and 3′ of cells when all components of the transfection were included, but not in controls missing any one component (Figure 4B). Although not quantified, band intensity appears greater in eGFP positive populations compared to nonsorted cells. Interestingly, light banding could also be observed in eGFP negative cell populations (Figure 4B). Sequencing of cloned junction amplicons from the eGFP positive populations showed that precise HMEJ is a frequent repair mechanism, but variants with imprecise HMEJ or NHEJ integration junctions were also observed (Figure 4C). Precise and imprecise HMEJ along with NHEJ insertions were also observed in eGFP negative cells by sequencing (Figure 4C). HMEJ and NHEJ junctions in the eGFP negative cells either indicate partial insertion events where the entire cassette was not integrated or cases where eGFP expression was silenced.

## Discussion

Here, we report significantly improved gene editing in porcine spermatogonia by demonstrating efficient site-specific indel generation and HDR from ssODN and plasmid cassette donors using the CRISPR/Cas9 RNP system. This is the first application of CRISPR/Cas9 in pig spermatogonia, and rates of editing by NHEJ were nearly double of what we reported previously using TALENs (Tang et al., 2018). Compared with somatic cells, spermatogonia, including SSCs, are more refractory to transfection (Zheng et al., 2017). SSCs are also very sensitive to double-strand breaks (DSBs) and are more prone to undergo apoptosis in response to DSBs than somatic cells (Zheng et al., 2018). The cell recovery after transfection when using CRISPR/Cas9 RNPs was greatly improved compared to transfection with TALEN plasmids, almost twofold. This difference in efficiency and cell recovery could be due to multiple factors. First, our highest editing rates using TALENs required delivery of 25–50 μg of TALEN expressing plasmid. This quantity is 10–20-fold more plasmid DNA than required to achieve a similar editing rate in pig fibroblasts under similar conditions (Carlson et al., 2012), and much greater than the 1.7 μg of plasmid used here as a template for HMEJ. This high quantity of plasmid could alone account for the lower cell recovery rates. Second, CRISPR/Cas9 RNPs are an active complex and do not rely on the cell’s transcriptional and translational machinery to produce active editing reagents. Last, optimal TALEN editing occurred in spermatogonia at 30°C where cell recovery was reduced. In contrast, CRISPR/Cas9 RNPs performed well at 37°C where cell recovery is at its highest.

The ability to use HDR to generate precise mutations greatly expands the versatility of SSC gene editing. Whereas attempts to stimulate HDR with TALENs had previously failed (unpublished), we were encouraged to revisit HDR considering the higher rates of cell editing and recovery with CRISPR/Cas9 RNPs. To our surprise, HDR editing using ssODNs was achieved at rates of 10–40%. As with our previous results in fibroblasts, insertional HDR edits were more efficient than edits that introduced SNPs (Tan et al., 2013), presumably by enhancing the stability of resulting edited alleles. This high rate of editing unlocks the potential to directly model dominant or gain of function alleles identified in humans in founder pigs produced by SSC editing, exemplified by our choice to engineer *HNF1a* and *INS* to model dominant forms of diabetes.

Single-stranded DNA templates longer than standard oligonucleotides (60–200 bases) are difficult and expensive to produce in the quantities required for HDR. This restricts the application of ssDNA to introduction of small alleles in the range of 1–150 base pairs. However, several biomedical applications benefit from introduction of transgeneses and/or gene replacements in a site-specific manner. Precisely integrated transgenes are useful for a diversity of applications such as cell reporting, cell-specific ablation, and immune modulation (Ruan et al., 2015; Carneiro D’Albuquerque et al., 2018). Our results demonstrate that as observed in pig fibroblasts (Wierson et al., 2020), HMEJ insertion is effective in porcine spermatogonia. Based on GFP expression, our integration rate of ∼8% is encouraging, but this is a relatively small (3 kb) expression cassette, and it will be interesting to determine if transgenes with much larger cargos can be introduced. The approaches established in the current study for efficient targeted genome editing in porcine spermatogonia have been used in other species and cell types. However, to our knowledge, this is the first example of CRISPR/Cas9 RNP-mediated HDR and HMEJ transgene insertion in primary spermatogonia of any species, further expanding the SSC editing toolbox.

Since germline-competent embryonic stem cells (ESCs) are not well established in pigs, the generation of engineered pigs currently relies on SCNT and zygote injection or electroporation. These established approaches require manipulation of embryos, which can result in chimerism, incomplete reprogramming of the somatic cell nuclei, abnormal fetal and placental development, or neonatal mortality. Due to their reliance on oocytes obtained from commercial pigs at slaughter, zygote injection is not applicable to smaller strains of pigs that are more suitable for biomedical research than large commercial breeds. The generation of pig models using gene-edited SSCs and germline stem transplantation is advantageous in that it avoids the production of mosaic mutant progeny, can be applied to diverse strains of pigs, and shortens the timeline to production of gene-edited spermatozoa (Tang et al., 2015). As the genetic change is introduced into the male germline just before the onset of spermatogenesis, the approach is more broadly applicable to disease models where gene dosage and epigenetics play a role. The production of rodent progeny with targeted genetic modifications following transplantation of gene-edited SSCs and *in vitro* fertilization or natural breeding has been achieved (Chapman et al., 2015; Sato et al., 2015; Wu et al., 2015).

In pig, cattle, sheep, and goats with functional immune systems, transplantation of germ cells including SSCs isolated from unrelated donors demonstrated that the recipient testes is immunotolerant, simplifying the approach by eliminating the need to identify and use genetically related donors or induce immune suppression before transplantation (Honaramooz et al., 2002, 2008; Herrid et al., 2006; Rodriguez-Sosa et al., 2006; Zeng et al., 2012, 2013). To improve the outcome of germ cell transplantation, effective chemical and radiological approaches to ablate a recipient’s endogenous SSCs and expand the availability of the stem cell niche for the transplanted SSCs to colonize have been developed in pigs (Honaramooz et al., 2005). Recently, pig models with genetically impaired spermatogenesis have been generated to overcome the drawbacks associated with chemical and radiological SSC ablation (Nicholls et al., 2019; Ciccarelli et al., 2020). We are optimistic that the high rates of editing reported here along with transplantation into germline ablated pigs will enable efficient production of gene edited founders.

We also recognize that success in germ cell gene-editing followed by transplantation is dependent on characteristics of isolated spermatogonia including purity and cell viability. To obtain a pure population of pig spermatogonia, we recently refined a differential plating protocol for pig germ cell enrichment (Sakib et al., 2019). Further enrichment was achieved using flow activated cell sorting and light scatter properties (Tang et al., 2018). These approaches allowed for efficient gene editing in primary spermatogonia in the current study. *In vitro* culture conditions that promote proliferation and long-term culture of mouse SSCs are well established facilitating the process of obtaining a highly enriched and robust population of SSCs for gene editing with engineered nucleases. Culture conditions for mouse germ cells have not translated to pig germ cell culture where proliferation and long-term culture remain limited. However, recent advances in improved culture systems for porcine spermatogonia (Zhang et al., 2017; Zheng et al., 2020) may allow for gene targeting in porcine germ cells at a larger scale.

A specific limitation of producing gene-edited animals by germline stem cell transplantation is that founder offspring will carry only one engineered allele. This limits the ability to directly produce biomedical animals where homozygosity is required to achieve a desired phenotype. However, there are numerous dominant disorders and biomedical applications that can be produced in the heterozygous state. The results reported here are critical to unlock the potential of dominant disease modeling or site-specific transgene integration, and represent an attractive alternative to SCNT or zygote manipulation for this purpose.

## Data Availability Statement

The raw data supporting the conclusions of this article will be made available by the authors, without undue reservation.

## Ethics Statement

The animal study was reviewed and approved by the Animal Care and Use Committee at the University of Calgary.

## Author Contributions

DW, SS, AB, NL, and LS designed and performed the experiments under the direction of DC and ID. All authors contributed to data analysis. SS, TG, DC, and ID wrote the manuscript.

## Conflict of Interest

DW, SS, TG, DC, and ID are employees and/or shareholders in Recombinetics, Inc., a company that commercializes gene editing in livestock. The remaining authors declare that the research was conducted in the absence of any commercial or financial relationships that could be construed as a potential conflict of interest.

## Supplementary Material

Supplementary Figure 1 Analysis of indel formation (NHEJ) in the *ROSA26* locus by Surveyor assay after editing with duplexed or single gRNAs at two different temperatures. Arrowheads indicate digested fragments (see Table 1).

## References

[B1] Armstrong L. Lako M. Dean W. Stojkovic M. (2006). Epigenetic modification is central to genome reprogramming in somatic cell nuclear transfer. *Stem Cells* 24 805–814. 10.1634/stemcells.2005-0350 16282443

[B2] Bae S. Park J. Kim J. S. (2014). Cas-OFFinder: A fast and versatile algorithm that searches for potential off-target sites of Cas9 RNA-guided endonucleases. *Bioinformatics* 30 1473–1475. 10.1093/bioinformatics/btu048 24463181 PMC4016707

[B3] Bonk A. J. Li R. Lai L. Ho Y. Liu Z. Samuel M. (2008). Aberrant DNA methylation in porcine in vitro-, parthenogenetic-, and somatic cell nuclear transfer-produced blastocysts. *Mol. Reprod Dev.* 75 250–264. 10.1002/mrd.20786 17595009 PMC2488202

[B4] Brinster R. L. Avarbock M. R. (1994). Germline transmission of donor haplotype following spermatogonial transplantation. *Proc. Natl. Acad. Sci.* 91 11303–11307. 10.1073/pnas.91.24.11303 7972054 PMC45219

[B5] Brinster R. L. Zimmermann J. W. (1994). Spermatogenesis following male germ-cell transplantation. *Proc. Natl. Acad. Sci. USA* 91 11298–11302. 10.1073/pnas.91.24.11298 7972053 PMC45218

[B6] Carlson D. F. Tan W. Lillico S. G. Stverakova D. Proudfoot C. Christian M. (2012). Efficient TALEN-mediated gene knockout in livestock. *Proc. Natl. Acad. Sci. USA* 109 17382–17387. 10.1073/pnas.1211446109 23027955 PMC3491456

[B7] Carneiro D’Albuquerque L. A. Reyes L. M. Estrada J. L. Wang Z. Y. Tector M. Tector A. J. (2018). CRISPR/Cas and recombinase-based human-to-pig orthotopic gene exchange for xenotransplantation. *J. Surg. Res.* 229 28–40. 10.1016/j.jss.2018.03.051 29937002

[B8] Chapman K. M. Medrano G. A. Jaichander P. Chaudhary J. Waits A. E. Nobrega M. A. (2015). Targeted germline modifications in rats using CRISPR/Cas9 and spermatogonial stem cells. *Cell Rep.* 10 1828–1835. 10.1016/j.celrep.2015.02.040 25772367 PMC4376630

[B9] Ciccarelli M. Giassetti M. I. Miao D. Oatley M. J. Robbins C. Lopez-Biladeau B. (2020). Donor-derived spermatogenesis following stem cell transplantation in sterile *NANOS2* knockout males. *Proc. Natl. Acad. Sci. U S A* 117 24195–24204. 10.1073/pnas.2010102117 32929012 PMC7533891

[B10] De Rooij D. G. (2001). Proliferation and differentiation of spermatogonial stem cells. *Reproduction* 121 347–354. 10.1530/rep.0.1210347 11226060

[B11] De Sousa P. A. King T. Harkness L. Young L. E. Walker S. K. Wilmut I. (2001). Evaluation of gestational deficiencies in cloned sheep fetuses and placentae. *Biol. Rep.* 65 23–30. 10.1095/biolreprod65.1.23 11420219

[B12] Guschin D. Y. Waite A. J. Katibah G. E. Miller J. C. Holmes M. C. Rebar E. J. (2010). A rapid and general assay for monitoring endogenous gene modification. *Meth. Mol. Biol.* 649 247–256. 10.1007/978-1-60761-753-2_15 20680839

[B13] Hamra F. K. Gatlin J. Chapman K. M. Grellhesl D. M. Garcia J. V. Hammer R. E. (2002). Production of transgenic rats by lentiviral transduction of male germ-line stem cells. *Proc. Natl. Acad. Sci. USA* 99 14931–14936. 10.1073/pnas.222561399 12391306 PMC137522

[B14] Herrid M. Vignarajan S. Davey R. Dobrinski I. Hill J. R. (2006). Successful transplantation of bovine testicular cells to heterologous recipients. *Reproduction* 132 617–624. 10.1530/rep.1.01125 17008473

[B15] Hill J. R. Schlafer D. H. Fisher P. J. Davies C. J. (2002). Abnormal expression of trophoblast major histocompatibility complex class I antigens in cloned bovine pregnancies is associated with a pronounced endometrial lymphocytic response. *Biol. Rep.* 67 55–63. 10.1095/biolreprod67.1.55 12079999

[B16] Honaramooz A. Behboodi E. Hausler C. L. Blash S. Ayres S. L. Azuma C. (2005). Depletion of endogenous germ cells in male pigs and goats in preparation for germ cell transplantation. *J. Androl.* 26 698–705. 10.2164/jandrol.05032 16291964 PMC1352318

[B17] Honaramooz A. Megee S. O. Dobrinski I. (2002). Germ cell transplantation in pigs. *Biol. Reprod.* 66 21–28. 11751259 10.1095/biolreprod66.1.21

[B18] Honaramooz A. Megee S. Zeng W. Destrempes M. M. Overton S. A. Luo J. (2008). Adeno-associated virus (AAV)-mediated transduction of male germ line stem cells results in transgene transmission after germ cell transplantation. *FASEB J.* 22 374–382. 10.1096/fj.07-8935com 17873102

[B19] Isakson S. H. Rizzardi A. E. Coutts A. W. Kirstein M. N. Fisher J. (2018). Genetically engineered minipigs model the major clinical features of human neurofibromatosis type 1. *Commun. Biol.* 1:158. 10.1038/s42003-018-0163-y 30302402 PMC6168575

[B20] Kleinwort K. J. H. Amann B. Hauck S. M. (2017). Retinopathy with central oedema in an INS (C94Y) transgenic pig model of long-term diabetes. *Diabetologia* 60 1541–1549. 10.1007/s00125-017-4290-7 28480495

[B21] Luo J. Megee S. Dobrinski I. (2009). Asymmetric distribution of UCH-L1 in spermatogonia is associated with maintenance and differentiation of spermatogonial stem cells. *J. Cell Physiol.* 220 460–468. 10.1002/jcp.21789 19388011 PMC2732714

[B22] Nicholls P. K. Schorle H. Naqvi S. Hu Y. C. Yuting H. Carmell A. (2019). Mammalian germ cells are determined after PGC colonization of the nascent gonad. *Proc. Natl. Sci. USA* 116 25677–25687. 10.1073/pnas.1910733116 31754036 PMC6925976

[B23] Orwig K. E. Avarbock M. R. Brinster R. L. (2002). Retrovirus-mediated modification of male germline stem cells in rats. *Biol. Reprod* 67 874–879. 10.1095/biolreprod.102.005538 12193397

[B24] Park J. Bae S. Kim J. S. (2015). Cas-Designer: A web-based tool for choice of CRISPR-Cas9 target sites. *Bioinformatics* 31 4014–4016. 26358729 10.1093/bioinformatics/btv537

[B25] Renner S. Braun-Reichhart C. Blutke A. Herbach N. Emrich D. Streckel E. (2013). Permanent neonatal diabetes in INS(C94Y) transgenic pigs. *Diabetes* 62 1505–1511. 10.2337/db12-1065 23274907 PMC3636654

[B26] Rodriguez-Sosa J. R. Dobson H. Hahnel A. (2006). Isolation and transplantation of spermatogonia in sheep. *Theriogenology* 66 2091–2103. 10.1016/j.theriogenology.2006.03.039 16870245

[B27] Rogers C. S. Stoltz D. A. Meyerholz D. K. Ostegaard L. S. Rokhlina T. Tafts P. J. (2009). Disruption of the CFTR gene produces a model of cystic fibrosis in newborn pigs. *Science* 321 1837–1841. 10.1126/science.1163600 18818360 PMC2570747

[B28] Ruan J. Li H. Xu K. Wu T. Wei J. Zhou R. (2015). Highly efficient CRISPR/Cas9-mediated transgene knockin at the H11 locus in pigs. *Sci. Rep.* 5 142–153. 10.1038/srep14253 PMC4585612 26381350

[B29] Sakib S. Yu Y. Voigt A. Ungrin M. Dobrinski I. (2019). Generation of Porcine Testicular Organoids with Testis Specific Architecture using Microwell Culture. *J. Vis. Exp.* 152:e60387. 10.3791/60387 31633676

[B30] Sato T. Sakuma T. Yokonishi T. Katagiri K. Kamimura S. Ogonuki N. (2015). Genome Editing in Mouse Spermatogonial Stem Cell Lines Using TALEN and Double-Nicking CRISPR/Cas9. *Stem Cell Rep.* 5 75–82. 10.1016/j.stemcr.2015.05.011 26095606 PMC4618438

[B31] Tan W. S. Carlson D. F. Walton M. W. Fahrenkrug S. C. Hackett P. B. (2012). Precision editing large animal genomes. *Adv. Genet.* 80 37–97. 10.1016/B978-0-12-404742-6.00002-8 23084873 PMC3683964

[B32] Tan W. Carlson D. F. Lancto C. A. Garbe C. A. Webster D. A. Hackett P. B. (2013). Efficient non-meiotic allele introgression in livestock using custom endonucleases. *Proc. Natl. Acad. Sci. USA* 110 16526–16531. 10.1073/pnas.1310478110 24014591 PMC3799378

[B33] Tang L. Bondareva A. Gonzalez R. Rodriguez-Sosa J. R. Carlson D. F. Webster D. (2018). TALEN-mediated gene targeting in porcine spermatogonia. *Mol. Reprod. Dev.* 85 250–261. 10.1002/mrd.22961 29393557 PMC6370346

[B34] Tang L. Gonzalez-Herrero R. Dobrinski I. (2015). Germline modification of domestic animals. *Anim. Reprod.* 12 93–104. 27390591 PMC4933526

[B35] Tian X. C. Park J. Bruno R. French R. Jiang L. Prather R. S. (2009). Altered gene expression in cloned piglets. *Reprod Fertil. Dev.* 21 60–66. 10.1071/RD08214 19152746

[B36] Wierson W. A. Welker J. M. Almeida M. P. Mann C. M. Webster D. A. Torrie M. E. (2020). Efficient targeted integration directed by short homology in zebrafish and mammalian cells. *eLife* 9:e539681–25. 10.7554/eLife.53968 32412410 PMC7228771

[B37] Wu Y. Zhou H. Fan X. Zhang Y. Zhang M. Wang Y. (2015). Correction of a genetic disease by CRISPR-Cas9-mediated gene editing in mouse spermatogonial stem cells. *Cell Res.* 25 67–79. 10.1038/cr.2014.160 25475058 PMC4650588

[B38] Zeng W. Tang L. Bondareva A. Honaramooz A. Tanco V. Dores C. (2013). Viral transduction of male germline stem cells results in transgene transmission after germ cell transplantation in pigs. *Biol. Reprod.* 88 1–9. 23221397 10.1095/biolreprod.112.104422 PMC4434942

[B39] Zeng W. Tang L. Bondareva A. Luo J. Megee S. O. Modelski M. (2012). Non-viral transfection of goat germline stem cells by nucleofection results in production of transgenic sperm after germ cell transplantation. *Mol. Reprod. Dev.* 79 255–261. 10.1002/mrd.22014 22231935 PMC3368892

[B40] Zhang P. Chen X. Zheng Y. Zhu J. Quin J. Lv Y. (2017). Long-term propagation of porcine undifferentiated spermatogonia. *Stem Cells Dev.* 26 1121–1131. 10.1089/scd.2017.0018 28474535 PMC5563923

[B41] Zheng Y. Feng T. Zhang P. Lei P. Li F. Zeng W. (2020). Establishment of cell lines with porcine spermatogonial stem cell properties. *J. Anim. Sci. Biotechnol.* 11:33 10.1186/s40104-00439-0 PMC7146966 32308978

[B42] Zheng Y. Jongejan A. Mulder C. L. Mastenbroek R. S Wang Y. (2017). Trivial role for NSMCE2during in vitro proliferation and differentiation of male germline stem cells. *Reproduction* 154 181–195. 10.1530/REP-17-0173 28576919

[B43] Zheng Y. Lei Q. Jongejan A. Mulder C. L. van Daalen SKM Mastenbroek S. (2018). The influence of retinoic acid-induced differentiation on the radiation response of male germline stem cells. *DNA Repair* 70 55–66. 10.1016/j.dnarep.2018.08.027 30179733 PMC6237089

