# Supplementary figures and images for: Targeted Gene Editing in Porcine Spermatogonia
Source: Front Genet. 2021 Jan 28;11:627673. doi: 10.3389/fgene.2020.627673 (PMC7876475; doi:10.3389/fgene.2020.627673)

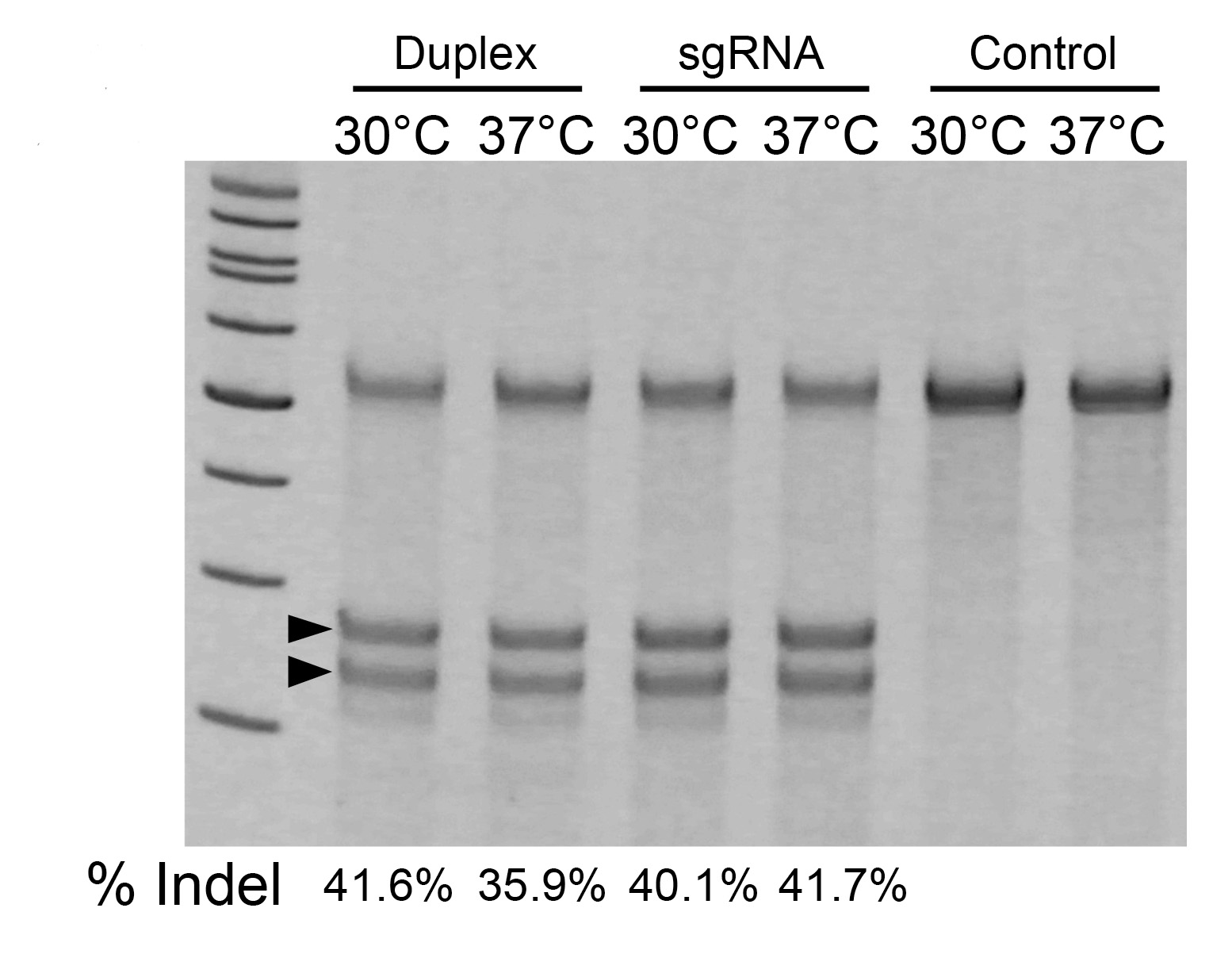

Supplement: Supplementary Figure 1 — Analysis of indel formation (NHEJ) in the ROSA26 locus by Surveyor assay after editing with duplexed or single gRNAs at two different temperatures. Arrowheads indicate digested fragments (see Table 1). [file Image_1.jpg]
