# Supplementary material for: Targeted Gene Editing in Porcine Spermatogonia
Source: Front Genet. 2021 Jan 28;11:627673. doi: 10.3389/fgene.2020.627673 (PMC7876475; doi:10.3389/fgene.2020.627673)
Supplement: Supplementary file 2 [file Table_1.docx]

**pHMEJ-*ROSA26*-UbEGFP plasmid containing repair cassette**

Underlined: Universal gRNA

Italics: Homology arms

Bold: Functional components of EGFP expression cassette

Sequence:

CTGGATCCAGATCCCTATACAGTTGAAGTCGGAAGTTTACATACACTTAAGTTGGAGTCATTAAAACTCGTTTTTCAACTACTCCACAAATTTCTTGTTAACAAACAATAGTTTTGGCAAGTCAGTTAGGACATCTACTTTGTGCATGACACAAGTCATTTTTCCAACAATTGTTTACAGACAGATTATTTCACTTATAATTCACTGTATCACAATTCCAGTGGGTCAGAAGTTTACATACACTAAGTTGACTGTGCCTTTAAACAGCTTGGAAAATTCCAGAAAATGATGGTCTCCCTATCGGGAGGCGTTCGGGCCACAGCGGTGAT*ATACCTTTATGGGAGTTCTCTGCTGCCTCCTTTTCCTAAGGACCGCCC*CTTCGATATCATTACTAGTTTAAACCTAGGATGCATACCGGTTACTGAGATC**TGGCCTCCGCGCCGGGTTTTGGCGCCTCCCGCGGGCGCCCCCCTCCTCACGGCGAGCGCTGCCACGTCAGACGAAGGGCGCAGGAGCGTCCTGATCCTTCCGCCCGGACGCTCAGGACAGCGGCCCGCTGCTCATAAGACTCGGCCTTAGAACCCCAGTATCAGCAGAAGGACATTTTAGGACGGGACTTGGGTGACTCTAGGGCACTGGTTTTCTTTCCAGAGAGCGGAACAGGCGAGGAAAAGTAGTCCCTTCTCGGCGATTCTGCGGAGGGATCTCCGTGGGGCGGTGAACGCCGATGATTATATAAGGACGCGCCGGGTGTGGCACAGCTAGTTCCGTCGCAGCCGGGATTTGGGTCGCGGTTCTTGTTTGTGGATCGCTGTGATCGTCACTTGGTGAGTAGCGGGCTGCTGGGCTGGCCGGGGCTTTCGTGGCCGCCGGGCCGCTCGGTGGGACGGAAGCGTGTGGAGAGACCGCCAAGGGCTGTAGTCTGGGTCCGCGAGCAAGGTTGCCCTGAACTGGGGGTTGGGGGGAGCGCAGCAAAATGGCGGCTGTTCCCGAGTCTTGAATGGAAGACGCTTGTGAGGCGGGCTGTGAGGTCGTTGAAACAAGGTGGGGGGCATGGTGGGCGGCAAGAACCCAAGGTCTTGAGGCCTTCGCTAATGCGGGAAAGCTCTTATTCGGGTGAGATGGGCTGTGGCACCATCTGGGGACCCTGACGTGAAGTTTGTCACTGACTGGAGAACTCGGTTTGTCGTCTGTTGCGGGGGCGGCAGTTATGGCGGTGCCGTTGGGCAGTGCACCCGTACCTTTGGGAGCGCGCGCCCTCGTCGTGTCGTGACGTCACCCGTTCTGTTGGCTTATAATGCAGGGTGGGGCCACCTGCCGGTAGGTGTGCGGTAGGCTTTTCTCCGTCGCAGGACGCAGGGTTCGGGCCTAGGGTAGGCTCTCCTGAATCGACAGGCGCCGGACCTCTGGTGAGGGGAGGGATAAGTGAGGCGTCAGTTTCTTTGGTCGGTTTTATGTACCTATCTTCTTAAGTAGCTGAAGCTCCGGTTTTGAACTATGCGCTCGGGGTTGGCGAGTGTGTTTTGTGAAGTTTTTTAGGCACCTTTTGAAATGTAATCATTTGGGTCAATATGTAATTTTCAGTGTTAGACTAGTAAATTGTCCGCTAAATTCTGGCCGTTTTTGGCTTTTTTGTTAGACGAGCTAGCTCGAGCCTGAATTCTACCATGGTGAGCAAGGGCGAGGAGCTGTTCACCGGGGTGGTGCCCATCCTGGTCGAGCTGGACGGCGACGTAAACGGCCACAAGTTCAGCGTGTCCGGCGAGGGCGAGGGCGATGCCACCTACGGCAAGCTGACCCTGAAGTTCATCTGCACCACCGGCAAGCTGCCCGTGCCCTGGCCCACCCTCGTGACCACCCTGACCTACGGCGTGCAGTGCTTCAGCCGCTACCCCGACCACATGAAGCAGCACGACTTCTTCAAGTCCGCCATGCCCGAAGGCTACGTCCAGGAGCGCACCATCTTCTTCAAGGACGACGGCAACTACAAGACCCGCGCCGAGGTGAAGTTCGAGGGCGACACCCTGGTGAACCGCATCGAGCTGAAGGGCATCGACTTCAAGGAGGACGGCAACATCCTGGGGCACAAGCTGGAGTACAACTACAACAGCCACAACGTCTATATCATGGCCGACAAGCAGAAGAACGGCATCAAGGTGAACTTCAAGATCCGCCACAACATCGAGGACGGCAGCGTGCAGCTCGCCGACCACTACCAGCAGAACACCCCCATCGGCGACGGCCCCGTGCTGCTGCCCGACAACCACTACCTGAGCACCCAGTCCGCCCTGAGCAAAGACCCCAACGAGAAGCGCGATCACATGGTCCTGCTGGAGTTCGTGACCGCCGCCGGGATCACTCTCGGCATGGACGAGCTGTACAAGTAAAGATCAATTCATTGATGAGTTTGGACAAACCACAACTAGAATGCAGTGAAAAAAATGCTTTATTTGTGAAATTTGTGATGCTATTGCTTTATTTGTAACCATTATAAGCTGCAATAAACAAGTTAACAACAACAATTGCATTCATTTTATGTTTCAGGTTCAGGGGGAGGTGTGGGAGGTTTTTTAAAGCAAGTAAAACCTCTACAAATGTGGT**CATTCAGCGATATCAAAGCTTCCCGGGAAG*TGGGCCTAGAAAAATCCCTCCCTCCCCCGCGATCTCGTCATCGCCTCC*GCTCCACTGTGGCCCGAACGCCTCCCAAAGGGAGACCAAGTTAAACAATTTAAAGGCAATGCTACCAAATACTAATTGAGTGTATGTAAACTTCTGACCCACTGGGAATGTGATGAAAGAAATAAAAGCTGAAATGAATCATTCTCTCTACTATTATTCTGATATTTCACATTCTTAAAATAAAGTGGTGATCCTAACTGACCTAAGACAGGGAATTTTTACTAGGATTAAATGTCAGGAATTGTGAAAAAGTGAGTTTAAATGTATTTGGCTAAGGTGTATGTAAACTTCCGACTTCAACTGTATAGGGATCTGGTACCATTTAAATCTGTTCCGCTTCCTCGCTCACTGACTCGCTGCGCTCGGTCGTTCGGCTGCGGCGAGCGGTATCAGCTCACTCAAAGGCGGTAATACGGTTATCCACAGAATCAGGGGATAACGCAGGAAAGAACATGTGAGCAAAAGGCCAGCAAAAGGCCAGGAACCGTAAAAAGGCCGCGTTGCTGGCGTTTTTCCATAGGCTCCGCCCCCCTGACGAGCATCACAAAAATCGACGCTCAAGTCAGAGGTGGCGAAACCCGACAGGACTATAAAGATACCAGGCGTTTCCCCCTGGAAGCTCCCTCGTGCGCTCTCCTGTTCCGACCCTGCCGCTTACCGGATACCTGTCCGCCTTTCTCCCTTCGGGAACCGTGGCGCTTTCTCATAGCTCACGCTGTAGGTATCTCAGTTCGGTGTAGGTCGTTCGCTCCAAGCTGGGCTGTGTGCACGAACCCCCCGTTCAGCCCGACCGCTGCGCCTTATCCGGTAACTATCGTCTTGAGTCCAACCCGGTAAGACACGACTTATCGCCACTGGCAGCAGCCACTGGTAACAGGATTAGCAGAGCGAGGTATGTAGGCGGTGCTACAGAGTTCTTGAAGTGGTGGCCTAACTACGGCTACACTAGAAGAACAGTATTTGGTATCTGCGCTCTGCTGAAGCCAGTTACCTTCGGAAAAAGAGTTGGTAGCTCTTGATCCGGCAAACAAACCACCGCTGGTAGCGGTGGTTTTTTTGTTTGCAAGCAGCAGATTACGCGCAGAAAAAAAGGATCTCAAGAAGATCCTTTGATCTTTTCTACGGGGTCTGACGCTCAGTGGAACGAAAACTCACGTTAAGGGATTTTGGTCATGAGATTATCAAAAAGGATCTTCACCTAGATCCTTTTTGCCAGTGTTACAACCAATTAACCAATTCTGATTAGAAAAACTCATCGAGCATCAAATGAAACTGCAATTTATTCATATCAGGATTATCAATACCATATTTTTGAAAAAGCCGTTTCTGTAATGAAGGAGAAAACTCACCGAGGCAGTTCCATAGGATGGCAAGATCCTGGTATCGGTCTGCGATTCCGACTCGTCCAACATCAATACAACCTATTAATTTCCCCTCGTCAAAAATAAGGTTATCAAGTGAGAAATCACCATGAGTGACGACTGAATCCGGTGAGAATGGCAAAAGTTTATGCATTTCTTTCCAGACTTGTTCAACAGGCCAGCCATTACGCTCGTCATCAAAATCACTCGCATCAACCAAACCGTTATTCATTCGTGATTGCGCCTGAGCGAGACGAAATACGCGATCGCTGTTAAAAGGACAATTACAAACAGGAATCGAATGCAACCGGCGCAGGAACACTGCCAGCGCATCAACAATATTTTCACCTGAATCAGGATATTCTTCTAATACCTGGAATGCTGTTTTTCCGGGGATCGCAGTGGTGAGTAACCATGCATCATCAGGAGTACGGATAAAATGCTTGATGGTCGGAAGAGGCATAAATTCCGTCAGCCAGTTTAGTCTGACCATCTCATCTGTAACATCATTGGCAACGCTACCTTTGCCATGTTTCAGAAACAACTCTGGCGCATCGGGCTTCCCATACAAGCGATAGATTGTCGCACCTGATTGCCCGACATTATCGCGAGCCCATTTATACCCATATAAATCAGCATCCATGTTGGAATTTAATCGCGGCCTCGACGTTTCCCGTTGAATATGGCTCATAACACCCCTTGTATTACTGTTTATGTAAGCAGACAGTTTTATTGTTCATGATGCAG
